# The Effects of Dominance on Leadership and Energetic Gain: A Dynamic Game between Pairs of Social Foragers

**DOI:** 10.1371/journal.pcbi.1002252

**Published:** 2011-10-20

**Authors:** Sean A. Rands

**Affiliations:** Centre for Behavioural Biology, School of Veterinary Science, University of Bristol, Bristol, United Kingdom; Kyoto University, Japan

## Abstract

Although social behaviour can bring many benefits to an individual, there are also costs that may be incurred whenever the members of a social group interact. The formation of dominance hierarchies could offer a means of reducing some of the costs of social interaction, but individuals within the hierarchy may end up paying differing costs dependent upon their position within the hierarchy. These differing interaction costs may therefore influence the behaviour of the group, as subordinate individuals may experience very different benefits and costs to dominants when the group is conducting a given behaviour. Here, a state-dependent dynamic game is described which considers a pair of social foragers where there is a set dominance relationship within the pair. The model considers the case where the subordinate member of the pair pays an interference cost when it and the dominant individual conduct specific pairs of behaviours together. The model demonstrates that if the subordinate individual pays these energetic costs when it interacts with the dominant individual, this has effects upon the behaviour of both subordinate and the dominant individuals. Including interaction costs increases the amount of foraging behaviour both individuals conduct, with the behaviour of the pair being driven by the subordinate individual. The subordinate will tend to be the lighter individual for longer periods of time when interaction costs are imposed. This supports earlier suggestions that lighter individuals should act as the decision-maker within the pair, giving leadership-like behaviours that are based upon energetic state. Pre-existing properties of individuals such as their dominance will be less important for determining which individual makes the decisions for the pair. This suggests that, even with strict behavioural hierarchies, identifying which individual is the dominant one is not sufficient for identifying which one is the leader.

## Author Summary

Dominance hierarchies could offer interacting animals a quick way to settle disputes without having to use too much effort. However, individuals may pay a price for acknowledging their position within the hierarchy, which could influence how they choose to behave within the group. Consequently, the actions of the group may be shaped by the effects of the hierarchy on each of the group’s members. I consider the behaviour of a pair that consists of a dominant and a subordinate individual, where the subordinate pays an energetic cost when it interacts with the dominant. I show that having to pay this cost affects the behaviour of the pair. I also demonstrate that, although a social hierarchy is imposed, the behaviour of the pair is not determined by the dominance relationship, but is instead influenced by the energetic reserves of the pair, where the decision-maker may just be whoever is the hungriest.

## Introduction

Animals can gain many benefits from associating in groups [1], but there are disadvantages that need to be considered as well. Competition for resources that cannot easily be shared equally (such as food, security, or access to mates) can lead to conflict between the members of the group [2]. Many species have social mechanisms for quickly or automatically resolving these conflict situations, such as the quick and definitive formation and maintenance of dominance relationships [3], [4]. Because group-living often means that individuals within a hierarchy are constantly interacting, these relationships can have long-term consequences upon the behaviours shown by individuals of different social standing, and therefore the effects of dominance need to be considered when we are interested in understanding the individual and collective behaviours of the group’s members. Ignoring the effects of these interactions could lead to us misunderstanding behavioural dynamics at the levels of the individual and of the group [5], [6].

Differing effects of dominance upon the behaviour of interacting individuals have been integrated into a number of theoretical studies. Some have concentrated upon how dominance affects the order of access to resources, where dominants may have priority or exclusive access to a foraging resource [7], [8]. Alternatively, dominant individuals may benefit during social foraging by reducing the degree of predation risk they experience during foraging by forcing subordinates into riskier positions [9]–[11] or more dangerous foraging periods during the day [12]. Other models have considered these imbalances in access to resources [13], as well as cases where dominance interactions lead to the spatial displacement of lower ranked individuals away from a patch [14], [15].

As well as effects upon foraging ability and group composition, living in social hierarchies can impose differing costs on individuals [16]–[20]. Rands *et al*. [6] considered an individual-based model consisting of a population made up of dominant and subordinate individuals, where all the individuals followed simple foraging rules when foraging, and where all individuals experienced the same energetic costs and gains during foraging, regardless of whether they were dominant or subordinate. However, subordinate individuals also experienced an additional cost when they foraged in close proximity to dominant individuals. This single additional cost had effects upon the movement behaviour and energetic stores of the subordinate individuals within the population. This single cost was considered to give a simple representation of a ‘socially mediated interference’ cost [21], [22], and considered the situation where a subordinate suffered if it foraged at the same time as a dominant individual (which could then be compared to a similar model where dominance costs weren’t considered [23]).

The way in which this socially mediated interference cost was implemented within the spatially-explicit individual-based framework of [6] involved making some broad assumptions about the rules that individuals use. As Rands [24], [25] discusses, making assumptions about rules within these models is useful, but we can greatly enhance the value of these rules if we are able to derive them from an even simpler set of assumptions. Therefore, in this paper I describe a state-dependent dynamic game model that explicitly considers the effects of several different socially-mediated costs upon the behaviour of a pair of socially foraging animals. I aim to show that imposing socially-mediated costs has effects upon the optimal behaviours of both the individual that is paying the costs (the subordinate), and in addition, upon the individual who is not directly paying these costs (the dominant). The dominant is instead being affected indirectly by these costs due to their effects upon the behaviour of the subordinate. In addition, I will also consider whether imposing costs of dominance mean that a dominant individual is the individual driving the behaviour of the pair.

## Methods

### Overview of model assumptions

The model followed here builds on the dynamic foraging game described by Rands *et al*. [26], [27]. In the simpler model described in [26], the decisions made by a pair of individuals are considered. The model uses dynamic programming techniques [28]–[32] to identify the optimal behaviours of a pair of animals, who are both characterised by possessing energy reserves (which defines their ‘state’) which change stochastically as a result of the actions that both individuals take over a series of consecutive decisions. Both members of the pair are able to accurately assess each other’s energetic reserves as well as their own, and their actions are informed by this information. During a period, each individual can choose to conduct one of two actions: either to rest or to forage for the entire period. Both actions incur an energetic cost which depletes the reserves of the individual, but foraging can also lead to the forager finding food (within a stochastic environment), meaning that, on average, it should see a net gain in its energetic reserves if it forages. Energetic reserves are important within the model: it is assumed that if they fall too low, an individual starves to death. It is also assumed that there is an upper limit to the capacity of the reserves, beyond which they cannot be increased further.

As well as the risk of starvation if an individual doesn’t forage, there is also the risk of predation, which depends on the actions of *both* individuals in the pair. If an individual choses to rest during a period, it incurs a low risk of being predated. If it forages at the same time as its colleague, it incurs a moderate risk of being predated (which could be through increased protection against predation from being in a small group, or enhanced detection, or simply a dilution of risk). If it forages on its own (whilst its colleague rests), it incurs the greatest risk of being predated. Therefore, there is a trade-off within this framework between being predated when foraging, and starving whilst resting. Rands *et al*. [26] demonstrate that these assumptions can be modelled using a stochastic dynamic game, and show that optimal policies can be calculated, which describe the optimal actions of an individual within a pair: the policies allow an individual to identify the suitable action to conduct given that it knows its own energetic reserves and those of its colleague at a given moment in time. Rands *et al*. [27] extend these models by considering what occurs when individuals are not identical in the costs they incur for conducting actions, or the amount of energy they gain during a period, or in the risks they face when conducting specific actions.

The model I describe here builds further on this framework. Although Rands *et al*. [27] considered possible differences between individuals in various parameters, they did not consider what could happen in a dominance interaction, where specific behavioural interactions between the two members of a pair incurred additional costs to one of the members (which I refer to as the ‘subordinate’), similar to the socially-mediated interference costs proposed in [6]. Note that I assume both that the dominance hierarchy has been decided by the pair members prior to the start of the period modelled, and that this hierarchy is adhered to throughout, with no further requirements to maintain it (see [33] for work considering the formation and maintenance of hierarchies). Here, I consider there to be four possible situations where an additional cost can be incurred by the subordinate:

- when both it and the individual who does not pay a cost (which I refer to as the ‘dominant’) are foraging together (which could for example be a proximity or vigilance cost, or simply a reduction in foraging efficiency due to direct competition from the dominant);
- when both the dominant and the subordinate are resting (which again could be a proximity cost, or perhaps an energetic cost due to the subordinate expending energy providing a service such as grooming to the dominant);
- when the subordinate is foraging on its own (which could be through social anxiety at not knowing where the dominant individual is, or through an increased cost of scanning for food, predators, or the dominant);
- when the subordinate is resting on its own (which again could be through social anxiety at not knowing where the dominant individual is).

In (*iii*) and (*iv*), I assume the dominant individual is conducting the opposite behaviour to the subordinate.

### Model details

The model considers a dynamic game between pair of players consisting of a dominant and a subordinate individual. This dynamic game builds on solution procedures outlined in [28] and [34], following a state-dependent framework as described in [28]–[31]. General computation methods build on the dynamic game framework for pairs of foragers, outlined in [26] and described in detail in [27], and the reader is referred to the latter for full details of the assumptions and the computational solution process, which are not repeated here. Unless described here, details are identical to those given in [27], and consequently I do not repeat any analysis for the effects of variables other than the socially-mediated costs that are introduced in this paper. To summarise the procedure described in [27] in a brief, using the assumptions about the effects of pair members’ actions as detailed in the overview above, an initial candidate strategy is assumed. This defines all possible actions that each individual should take, given that it knows its own energetic reserves and those of its colleague at a moment in time. Assuming one of the pair members is using the current candidate strategy, a best response can be calculated for its colleague using dynamic programming. To do this, I make an additional initial assumption about how energetic state relates to fitness (where fitness is used as a common currency to compare all possible actions [35]), but this initial assumption about how fitness relates to state is rendered unimportant through strong backwards convergence [28]. Once an optimal response strategy to the current population strategy has been identified, the best response to that strategy could be calculated, and then the best response to that, and so forth, with the aim of identifying an evolutionarily stable strategy (ESS). However, it is difficult to iterate to an ESS using this direct route (*e.g.* [36], [37]), and I instead used a error-making approach [34], where the candidate strategy is updated at each iterative step by combining the previous candidate strategy with the newly identified best response strategy (weighting the new candidate strategy strongly towards the previous candidate strategy). Using this technique, an ESS is identified through an iterative computational process. For finer detail of the assumptions, please see [26] and [27].

Where the current model differs from that presented in [27] is in the detail of the function denoted *H_i_*(*x_i_*, *x_j_*, *t*; *u_i_*, *u_j_*, **π**), which defines the probability that an individual of type *i* who is alive at the start of time step *t*, in state *x_i_* (>0), paired with a living colleague of corresponding type *j* in state *x_j_* (>0) who follows a strategy defined by the candidate strategy **π**, will survive until the start of the final time step *T*, if it adopts action *u_i_* and its colleague adopts action *u_j_* in the current time step (assuming that the focal individual *i* thereafter behaves so as to maximise its chances of surviving until time step *T*, taking into account errors in decision making). Note that, as with the model described in [27], the candidate strategy **π** encompasses the candidate responses of both subordinate and dominant individuals within the population (which means that it defines the current ‘best’ action that an individual should take given that it knows its own energy reserves, and those of its colleague: the modelling process considers all possible state combinations of energy reserves for both dominant and subordinate individual, and the candidate strategy therefore includes current ‘best’ actions for all of these).

In order to consider the effects of dominance on behaviour, I replace the *H_i_*(*x_i_*, *x_j_*, *t*; *u_i_*, *u_j_*, **π**) function described in [27] with the following set of definitions, which are dependent upon whether the focal individual is dominant or subordinate, and assume that both individuals in a pair are alive at the moment the decision is made (note that the associated functions that describe what occurs when only the focal individual is alive at the decision point, or that describe what happens if no individuals are alive at the decision point, are identical to those presented in [27], and are therefore not described here). Throughout, terms relevant to subordinates are denoted with a subscript *s*, and terms relevant to dominants are denoted with a subscript *d*.

If the individual is dominant, I assume
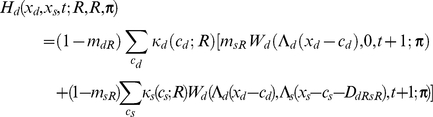


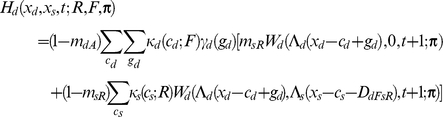


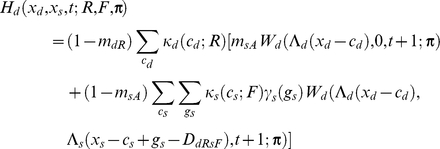


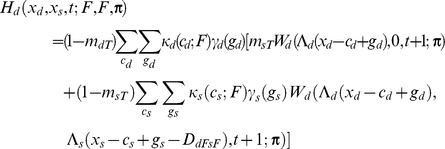



If the focal individual is subordinate, I instead assume
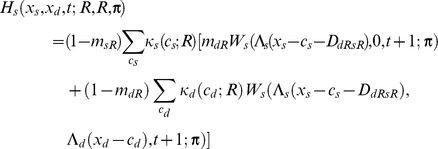


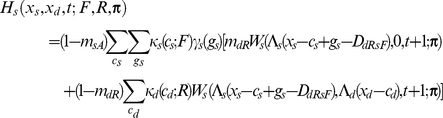


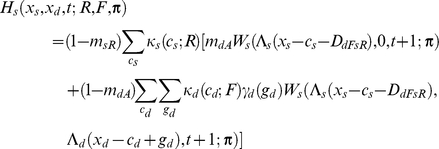


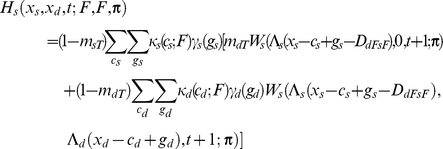



Apart from the novel interference cost terms (described below), terminology here follows [27], and is only briefly summarised here. An individual of type *a* has a probability *m_aR_* of being predated if it is resting, *m_aA_* if it is foraging alone, and *m_aT_* if it is foraging with its colleague. The function *W_a_*(*x_a_*,*x_b_*,*t*;***π***) is the fitness at time *t* of an individual of type *a* with energy reserves *x_a_*, and whose colleague has energy reserves *x_b_*, assuming that both individuals follow policy **π** from that point forward in time. Λ*_a_*(*x*) defines a ‘chop’ function for an individual of type *a* as defined in [30], where Λ*_a_*(*x*)  =  min(*S_a_*,max(*x*,0)), and *S_a_* is the maximum state value possible for an individual of type *a*. *κ_a_*(*c_a_*;*u*) denotes the probability that an individual of type *a* spends *c_a_* state units of energy during a period if it conducts action *u*, and *γ_a_*(*g_a_*) denotes the probability that it gains *g_a_* state units of energy during the period if it forages. Both energetic costs and gains were represented within the current model using the same functions described in [27], where the probabilities defined followed a discretised distribution based on normal distributions with defined means and standard deviations.

For simplicity, both dominant and subordinate individual were assumed to have identical probabilities of incurring given gains and costs, and share identical predation risks when conducting particular activities (so *m_dT_*  =  *m_sT_*, *etc*.). The only difference considered between them was in the extra cost paid by the subordinate individual when it was conducting a paired behaviour that incurred extra energetic costs. These energetic costs are denoted *D_dRsR_*, *D_dFsR_*, *D_dRsF_* and *D_dFsF_*, which represent the extra energetic cost (in state units) incurred for the four possible pairs of behaviours (where the general form *D_dUsV_* represents the cost paid by the subordinate when the dominant conducted action *U* and the subordinate conducted action *V*). The dominant is not expected to pay any extra costs for its actions dependent upon the behaviour of the subordinate.

### Summary statistics

Once stable behavioural policies for subordinate and dominant individuals had been identified, forward iterations using Markov chain processes [29] were then used to calculate the distribution of a pair’s states within a stable population. Again, full details of the process and assumptions made follow those described in [27]. Having identified a stable distribution of paired states, I then calculated the following summary statistics:

#### Individual behaviours

Having identified both the optimal policies for dominant and subordinate members of a pair, as well as the stable distribution of paired energetic reserves within the population, I was therefore able to calculate the proportion of the dominant and the subordinate population that would be foraging during a period.

#### Paired behaviours

Similarly, knowing stable paired states and policies also allowed us to calculate the proportions of the population where both members of a pair foraged during a period (*p_FF_*), where both members of a pair rested during a period (*p_RR_*), where the dominant foraged and the subordinate rested (*p_FR_*), or where the dominant rested and the subordinate foraged (*p_RF_*).

#### Synchrony coefficient

Having calculated the proportions of the population conducting the four types of paired behaviour, I quantified the amount of behavioural synchronisation during a period as (*p_RR_* ⋅ *p_FF_* - *p_RF_* ⋅ *p_FR_*)/(*p_RR_* ⋅ *p_FF_* + *p_RF_* ⋅ *p_FR_*). Note this term is referred to in [26] and [27] as *D*′, but I avoid using this notation here to avoid confusion with the cost of dominance used in the current model.

#### Independence of action

Rands & Johnstone [38] describe two similar methods for quantifying the degree of dependence that paired individuals have upon knowing the state of a colleague in order to be able to conduct the optimal behaviour: this demonstrates whether synchronisation of behaviour (or lack of it) can only come about through pair members having to track each others’ state, or whether any observed synchronisation is merely a by-product of tracking both individuals’ behaviour in isolation to their colleagues. Here, I use the *S* statistic described in [38], which provides a more naturalistic information-driven statistic than the *C* statistic described in the same paper.

#### Repeatability of behaviours

Given that a dominant or a subordinate can choose to conduct one behaviour during a period, the stable population and policies calculated above could be used to calculate the proportion of the population where the individual then conducts the same behaviour in the following period. Tracking repetition over time, I also calculated the repeatability of paired behaviours: this was taken to be the mean number of periods until at least 50% of the population had conducted at least one different pair of behaviours to that which they were engaged in at the initial period recorded.

#### Energetic reserves

he mean energetic reserves of the dominant and the subordinate individuals were calculated from the stable population distributions calculated above.

#### Repeatability of difference in energetic reserves

Due to the stochastic nature of the system, it was possible that both the dominant and the subordinate individual could end up having the highest energetic reserves. I calculated the mean length of time that dominants or subordinates maintained the rôle of ‘heaviest individual’ within the pair.

### Parameter exploration

I explored the effects of the costs by randomly generating 1,000 sets of other model parameters, and then calculating optimal policies and population distributions for all possible combinations of the four socially-mediated interference costs. Table 1 describes the parameters used within this model, including the use of randomisation to generate differences between parameter sets. For each set of parameters, I considered the sixteen possible scenarios where *D_dRsR_*, *D_dFsR_*, *D_dRsF_* and *D_dFsF_* could each take a value of either 0 or 1 state units, representing a full spectrum of cases where there was a potential cost to be paid by a subordinate individual dependent upon the actions of the dominant member of the pair. After calculating policies and stable population distributions, summary statistics were calculated for each of these sixteen possible scenarios, and exploratory analyses were conducted as detailed below.

**Table 1:** Parameters used in model, with values used for model exploration.

Variable	Description	Value
*c_max_*	Largest cost possible	4 state units
*D_dFsF_*	Extra energetic cost paid by subordinate when both it and the dominant are foraging	0 or 1
*D_dFsR_*	Extra energetic cost paid by subordinate when it is resting and the dominant is foraging	0 or 1
*D_dRsF_*	Extra energetic cost paid by subordinate when it is foraging and the dominant is resting	0 or 1
*D_dRsR_*	Extra energetic cost paid by subordinate when both it and the dominant are resting	0 or 1
*g_max_*	Maximum gain during a period	6 state units
*k*	Error in decision making	0.0000001
*λ*	Population adjustment constant	0.1
*m_A_*	Predation risk when foraging alone	exp(-25*r*_1_)
*m_R_*	Predation risk when resting	*m_T_*(1-(*r*_2_)^2^)
*m_T_*	Predation risk when foraging together	*m_A_*(1-(*r*_3_)^2^)
*µ_F_*	Mean cost of foraging	(*r*_4_ ⋅ *ν*) state units
*µ_R_*	Mean cost of resting	(*r*_5_ ⋅ *µ_F_*) state units
*ν*	Mean gain from foraging	(4 *r*_6_ + 1) state units
*ψ*	s.d. of energetic gain when foraging	(0.5)^0.5^ state units
*S*	Maximum state possible	40 state units
*σ_F_*	s.d. of energetic gain when foraging	(0.5)^0.5^ state units
*σ_R_*	s.d. of energetic gain when resting	(0.5)^0.5^ state units

Where these are not discussed in the text, refer to [27] for full clarification of their purpose. Note also that, with the exception of *D_dFsF_*, *D_dFsF_*, *D_dFsF_* and *D_dFsF_* (all of which are only experienced by the subordinate), all values are assumed equal for subordinate and dominant individuals. To generate a set of parameters for use within the simulations, six values *r_1_*, *r_2_*, *r_3_*, *r_4_*, *r_5_* and *r_6_* were randomly and independently sampled from a uniform distribution with the range (0, 1).

### Analyses of summary statistics

The model considers four possible interference costs to a subordinate individual, all of which could potentially have a separate effect upon its energetic turnover during a period dependent upon the behaviour of the pair. Therefore, I was interested in the interactions of the costs, as well as each of the costs themselves. To explore this, standard analysis of variance was used to generate *F* values for the four costs and the eleven possible interactions involving two, three or all four of these. The distribution of the results generated would not fit the standard assumptions necessary for ANOVA, and so I used resampling methods to identify critical *F* values, following recommendations in [39]. Because I was potentially interested in the effects of interactions, I would have been unable to generate resampled critical *F* values by the random assortment of results such that the untested costs or interactions were kept correctly assorted. However, my sample population of results was large, and I therefore generated resampled critical *F* values by freely permuting my entire dataset without restriction, following recommendations in [39]. For each, I used *R* 2.12.1 [40] to permute the entire dataset without replacement 50,000 times, harvesting the fifteen *F* values for an ANOVA conducted on each permuted set, and used the *quantile* function within *R* to identify the value of the 95% quantile values.

## Results

The statistical model considered includes two-, three- and four-way interactions, as these were biologically feasible within the framework considered. The results of these interactions are presented in full in Tables 2 and 3 for completeness, but only interesting relationships are discussed within the results section, as many of the relationships seen (especially for the three- and four-way interactions) were complex and difficult to interpret. All *F* values, along with the *F* values critical for demonstrating *p*<0.05 that were calculated by resampling, are reproduced in Supporting and S2.

**Table 2:** The effects of systematically changing dominance costs on the foraging behaviour of a pair.

Subordinate pays extra cost when	proportion of time dominant forages	proportion of time subordinate forages	proportion of time both players forage	proportion of time dominant forages, subordinate rests	proportion of time dominant rests, subordinate forages	proportion of time both players rest	synchrony coefficient	*S*
both players forage (FF)	+	+	+	–	+	–	–	–
the dominant forages, and the subordinate rests (FR)	NS	+	+	–	+	–	+	NS
the dominant rests, and the subordinate forages (RF)	+	+	+	–	+	–	+	NS
both players rest (RR)	+	+	+	–	+	–	–	+
**interaction terms**								
FF × FR	NS	+	+	*	NS	NS	–	NS
FF × RF	+	+	+	*	NS	–	–	–
FR × RF	NS	+	+	–	+	–	*	NS
FF × RR	+	+	+	NS	+	–	–	–
FR × RR	*	+	+	NS	NS	–	*	*
RF × RR	*	+	+	NS	NS	–	+	*
FF × FR × RF	*	NS	NS	*	*	NS	*	NS
FF × FR × RR	NS	*	*	*	NS	NS	*	NS
FF × RF × RR	*	*	*	*	*	*	NS	NS
FR × RF × RR	*	*	*	NS	NS	*	*	NS
FF × FR × RF × RR	*	*	NS	NS	NS	*	*	NS

This table reports the significance and direction of change for these result sets, based on ANOVA models containing all four costs of dominance and all possible interactions between these costs. Assuming a significance term of *p*<0.05, ‘+’ indicates that there was a significant increase in the behavioural measure when a cost was increased, or, in the case of a two-way interaction, increasing both terms led to an increase in the behavioural measure. ‘–’ indicates a similar decrease. ‘*’ indicates that an interaction term was significant, but did not follow a simple pattern of either both terms leading to an increase or to a decrease. ‘NS’ indicates that the measure was not significant (*p*≥0.05). Full statistical details are presented in Supporting Table S1.

**Table 3:** The effects of systematically changing dominance costs on history and energy reserves of a pair.

Subordinate pays extra cost when	likelihood dominant repeats behaviour	likelihood subordinate repeats behaviour	length of time a paired behaviour is repeated	energetic reserves of dominant	energetic reserves of subordinate	length of time dominant heaviest	length of time subordinate heaviest
both players forage (FF)	+	+	NS	+	–	NS	–
the dominant forages, and the subordinate rests (FR)	NS	+	+	+	+	–	+
the dominant rests, and the subordinate forages (RF)	NS	+	+	+	–	+	–
both players rest (RR)	–	–	–	+	–	NS	–
**interaction terms**							
FF × FR	NS	NS	NS	+	–	*	NS
FF × RF	*	+	*	NS	–	*	NS
FR × RF	NS	+	NS	+	+	*	NS
FF × RR	+	+	NS	NS	NS	*	–
FR × RR	NS	+	NS	NS	+	–	*
RF × RR	NS	+	NS	NS	–	+	NS
FF × FR × RF	NS	NS	NS	NS	NS	NS	NS
FF × FR × RR	*	*	NS	*	NS	NS	*
FF × RF × RR	NS	*	NS	NS	NS	NS	NS
FR × RF × RR	NS	NS	NS	NS	NS	NS	NS
FF × FR × RF × RR	NS	*	NS	NS	NS	NS	NS

See Table 2 for an explanation of the terminology used. Full statistical details are presented in Supporting Table S2.

### Individual behaviours

All individual increases in dominance cost led to an increase in the amount of foraging behaviour shown by the subordinate (Table 2) – there were also significant interactions between paired costs (and most three- or four-way interactions), although in all cases adding a cost led to an increase in foraging behaviour. Increasing the costs experienced by the subordinate led to increases in most of the individual foraging behaviour shown by the dominant, except for the case where the subordinate only experienced costs when it was resting and the dominant was foraging, which is likely to be a situation when the dominant is not going to be affected by the actions of the subordinate too much.

### Paired behaviours

The increases in individual foraging behaviour were also echoed in the paired behaviours (Table 2). Considering all the single costs within the statistical model, increasing any of the costs experienced by the subordinate individual led to an increase in its foraging behaviour, and a decrease in its resting behaviour. The fact that the direction of change is dictated solely by the subordinate individual suggests that the action of the dominant individual is being driven primarily by its foraging partner.

Paired costs led to increases in both individuals foraging together, and (apart from the case where the subordinate always paid a cost when the dominant was foraging), decreases in resting together. The paired interactions when the members of the pair were conducting differing behaviours were mostly non-significant, although there were increases in cases where the dominant rested and the subordinate foraged when the costs experienced by the subordinate occurred when the pair differed in their behaviour.

### Synchrony coefficient

As would be expected, pairs become more synchronised when there are costs involved with not being paired, and become less synchronised when there are costs to conducting the same action as each other (Table 2). Considering this alongside the paired behaviour results suggests that although the subordinate is driving the behaviours of the pair, its own actions are therefore partially dictated by the costs that it pays. Note also that this measure (with similar reasoning for the following *S* statistic) does not discriminate between resting together and foraging together. Therefore, an increase in only one of these paired behaviours may not lead to a corresponding increase in the general level of synchronisation within the pair.

### Independence of action

The *S* statistic decreased in response to a dominance cost when foraging together, and decreased when resting together (Table 2). Resting alone doesn’t incur any more risk of predation than when resting together, and it is therefore feasible that as resting together becomes more costly to the subordinate, it should therefore become more dependent upon the state of its colleague dictating its actions (in this case, avoiding resting together), leading to the decrease in synchrony shown by the synchrony coefficient. The decrease in dependence with an increasing cost of foraging together is echoed in the observation that the subordinate individual should be increasing its foraging regardless of the actions of the dominant.

### Repeatability of behaviours

Both the dominant and subordinate individuals tended to increase their repetition of behaviour when there was an extra cost to the subordinate of foraging at the same time as the dominant (Table 3). This is likely to be an effect of foraging being highly synchronised: the subordinate has to forage at the same time as the dominant, and consequently needs the pair to spend more time foraging than the dominant in order to fund the energy it spends (especially, but counterintuitively, whilst foraging). Both individuals tended to reduce their repetition of behaviour when there was an extra cost to the subordinate of resting together. This is likely to be due to the reduction in the amount of time that the subordinate rests overall – an increased likelihood of foraging suggests that a pair of individuals will be swapping between different pairs of behaviours, and is demonstrated in a similar reduction in the mean length of time that pairs of individuals repeated a paired behaviour (Table 3).

The subordinate individual also tended to repeat its own behaviour more often when there was a dominance cost associated with conducting the opposite behaviour to the dominant individual (note here that this means an overall increase in the subordinate repeating a behaviour irrespective of what the dominant is doing, rather than a statement that the subordinate is increasing conducting the opposite behaviour to the dominant). Most of the interactions shown for the subordinate individual also indicate a positive trend. Paired behaviours were also repeated more often when these costs were incurred. These increases are likely to be due to the increase in synchronisation behaviour seen when there is an extra cost to being non-synchronised.

### Energetic reserves

As would be expected, incurring an extra cost of dominance to the subordinate meant that its energetic reserves tended to be reduced (Table 3). This was not the case where there was a dominance cost to the subordinate when it rested and the dominant foraged, which may be due to an increase in the subordinate tending to forage in order to avoid this cost. Regardless of which sort of cost was imposed on the subordinate, the dominant tended to gain energetic reserves when there was a cost, which ties in with the increase in subordinate foraging behaviour and corresponding synchronisation by the dominant individual.

### Repeatability of difference in energetic reserves

The length of time that the subordinate remained heaviest (when it managed to reach that state of being) was in most cases reduced by imposing a cost of dominance (Table 3). The exception to this followed a similar pattern to the energetic reserves, where imposing a cost when the subordinate rested and the dominant foraged tended to lead to an increase in the length of time that the subordinate remained heaviest. Again, this is likely to be due to the subordinate increasing the amount of time it forages in response to this cost, therefore leading to an increase in its reserves. Increasing the length of time the subordinate individual remained heaviest should logically lead to a decrease in the length of time that the dominant individual remained heaviest, and *vice versa*. This trend was seen, but was only significant for the situations where the subordinate’s costs were paid for conducting the opposite behaviour to the dominant individual.

## Discussion

This model demonstrates that if a subordinate pays energetic costs when it interacts with a dominant individual, this has distinct effects upon the behaviour that it shows, and subsequently it affects the behaviour of the interacting dominant individual. Considered independently, both individuals tended to increase the amount of foraging behaviour they conducted when there were interaction costs. Considered together, the behaviour of the pair was driven by the subordinate individual. Costs imposed when the subordinate forages tend to increase paired foraging behaviour.

In the model presented here, the subordinate individual tended to be the lighter individual for longer periods of time when interaction costs were imposed. This lends support to the suggestion that the lighter individual acts as the decision-making ‘pace-maker’ of the group [26], [27], giving leadership-like behaviours that are based upon state [24], rather than specific pre-existing properties of individuals such as their dominance level [41] or tendency towards leadership [42]. As Rands et al. [27] discuss, consistent leadership behaviour can be a property of individuals with a higher metabolic requirement (such as in lactating female zebras [43]). Therefore, although a dominance relationship exists in the pairs modelled, the behaviour of the pair is determined by an individual whose identity emerges from the interaction between the pair, rather than being strictly set by which individual is dominant to which. This ‘leadership’ status should also be transient within the pair, with both the dominant and the subordinate individual taking it in turns to be lightest and thus determine the actions of the pair. Within the model, imposing most sorts of interaction cost leads to a reduction in the reserves of the subordinate individual, leading in turn to it remaining heaviest for less consecutive periods of time. This means that imposing a cost of interaction should lead to the subordinate individual becoming the decision-maker more often. Of course, it should be noted that although there are examples where subordinates tend to be the ones gaining the most energy reserves (*e.g.* [10], [44], [45]), there are also many empirical examples where dominant individuals tend to be both the decision-makers and the ones gaining the most food (*e.g.* [46]–[49]).

Therefore, imposing direct energetic costs of dominance should lead to effects upon the paired behaviour of foragers, lending support to the rules proposed for larger groups by Rands *et al*. [6]. Many of the costs of dominance are not directly energetic [9], [50]–[55]. Although these rules are a relatively simple representation of a possible cost, the idea of behaviourally-mediated interaction costs has biological merit. Differences may exist in energetic expenditure between individuals of different social ranks, as has been demonstrated in fish [18] and birds [19], [20] (but see [56]). Mass gain may differ between individuals of different ranks, even if they appear to show equal feeding rates [57], [58]. These differences in mass gain by individuals of differing social status may be due to differences in digestive ability [59], [60], or simply a behavioural difference in the amount of time spent foraging [52], [61] – both of which could be represented by the cost modelled here. Individuals could also be behaviourally mediating the costs that they pay in interactions [54], such as subordinates taking a lower share of resources when social dominance exists as a means of mediating the behavioural interaction. Lindström *et al*. [54] discuss whether a larger body mass could mean that individuals are better at mediating these costs. The model presented here only considers a difference in costs spent during activities, but could be extended to consider individuals with very different metabolic requirements, in a similar manner to the model presented by Rands *et al.* [27]. However, it is likely that a larger scale dominance model including differences between individuals would yield complex relationships that would not be simple to describe from a qualitative perspective, and should maybe be reserved for systems where some amount of parameterisation is possible.

Furthermore, the current model makes a simplifying assumption by assuming that the subordinate paid additional costs (and indeed, being subordinate is solely defined by paying these costs within the model). We could conceivably see a situation where the dominant individual also pays additional costs for being dominant. If these costs are less than those paid by the subordinate, these could simply be subsumed into the general metabolic costs paid by individuals, giving us a similar model structure to that described. However, if the dominant and the subordinate individual paid different levels of cost for different behaviours such that both paid more than the other for at least one of the four behavioural pairs, then this would be a situation not covered within the current model. For example, we could imagine a situation where the dominant paid the higher metabolic cost when foraging at the same time as the subordinate (such as through having to be aware of the subordinate’s foraging actions, and through expending energy in forcing the subordinate away from resources), whilst the subordinate could show a higher metabolic cost than the dominant when it was foraging on its own (such as through raising vigilance levels to spot both predators and in anticipation of the currently absent dominant individual). In this hypothetical example, the current model is not sufficient, and an extended version would need to be considered where costs to the dominant individual are also modelled. I would suggest that this exercise might be useful if exact predictions are needed for a well-defined system (such as tying the model in with an empirical system), but investigating a more general model would be unlikely to yield more tangible results than described in the simpler model I present here.

As well as behavioural interactions leading to subordinates gaining less energy during an interaction, physiological processes may also mean that they spend more energy, and could be mediated hormonally, such as through stress responses by individuals. Studies on many species have demonstrated that social stress and dominance interactions have effects upon body mass and composition [9], . Hierarchy rank and measures of stress typically depend on the social conditions experienced by animals, and whether there have been recent changes in how the social structure is organised [68]. Stress, as measured by levels of hormones such as glucocorticoids, shows no obvious relationship with dominance rank [69], although there are some correlations with species social system [70]. Stress has subtle short- and long-term effects upon an individual’s physiology, and care would be needed to catch these effects within a dynamic game, although it has been demonstrated that stress can be successfully captured using a state-dependent approach [71]. Again, careful parameterisation is necessary, but could be very useful for extending the rules suggested here to larger models considering complex social interactions (such as [6], [23], [72]–[74]), enhancing predictions about social behaviour and interactions.

## Supporting Information

Table S1 ***F*** **values and significance terms for results presented in** **Table 2**. In each cell, the first number is the *F*_1,11566_ value obtained from a fully-crossed ANOVA model that the fifteen terms given. The second number (in parentheses) is the critical *F*_(*p*<0.05)_ value estimated from resampling, above which a significance level of *p*<0.05 is assumed.
(DOCX)

Table S2 ***F*** **values and significance terms for results presented in** **Table 3**. Data are presented in an identical format to Supporting Table S1.
(DOCX)
