# Supplementary material for: The Effects of Dominance on Leadership and Energetic Gain: A Dynamic Game between Pairs of Social Foragers
Source: PLoS Comput Biol. 2011 Oct 20;7(10):e1002252. doi: 10.1371/journal.pcbi.1002252 (PMC3197661; doi:10.1371/journal.pcbi.1002252)
Supplement: Table S1 — F values and significance terms for results presented in Table 2 . In each cell, the first number is the F 1,11566 value obtained from a fully-crossed ANOVA model that the fifteen terms given. The second number (in parentheses) is the critical F (p<0.05) value estimated from resampling, above which a significance level of p<0.05 is assumed. (DOCX) [file pcbi.1002252.s001.docx]

| **Subordinate pays extra cost when** | **proportion of time dominant forages** | **proportion of time subordinate forages** | **proportion of time both players forage** | **proportion of time dominant forages, subordinate rests** | **proportion of time dominant rests, subordinate forages** | **proportion of time both players rest** | **synchrony coefficient** | ***S*** |
| --- | --- | --- | --- | --- | --- | --- | --- | --- |
| both players forage (FF) | 101.48 (3.85) | 775.03 (3.77) | 120.44 (3.85) | 11.93 (3.83) | 944.53 (3.85) | 683.46 (3.85) | 454.77 (3.85) | 210.66 (3.79) |
| the dominant forages, and the subordinate rests (FR) | 0.83 (3.85) | 234.93 (3.81) | 124.97 (3.89) | 511.68 (3.84) | 60.54 (3.81) | 26.22 (3.77) | 13.30 (3.87) | 1.39 (3.88) |
| the dominant rests, and the subordinate forages (RF) | 4.47 (3.83) | 156.85 (3.84) | 89.69 (3.86) | 269.75 (3.80) | 33.17 (3.87) | 26.07 (3.86) | 148.48 (3.87) | <0.01 (3.80) |
| both players rest (RR) | 281.47 (3.87) | 948.87 (3.90) | 296.18 (3.83) | 11.66 (3.84) | 624.53 (3.80) | 847.33 (3.83) | 168.43 (3.81) | 104.47 (3.82) |
| **interaction terms** |  |  |  |  |  |  |  |  |
| FF × FR | 0.17 (3.83) | 16.56 (3.78) | 18.90 (3.88) | 75.41 (3.82) | 0.15 (3.82) | 0.03 (3.88) | 17.88 (3.84) | 3.33 (3.88) |
| FF × RF | 19.06 (3.87) | 63.15 (3.77) | 58.64 (3.93) | 61.64 (3.82) | 0.52 (3.84) | 19.27 (3.83) | 40.89 (3.88) | 8.51 (3.86) |
| FR × RF | 0.50 (3.80) | 53.45 (3.83) | 23.93 (3.89) | 85.83 (3.85) | 20.21 (3.87) | 9.77 (3.77) | 16.22 (3.84) | 1.06 (3.83) |
| FF × RR | 16.81 (3.88) | 43.15 (3.83) | 19.70 (3.82) | 1.81 (3.82) | 15.69 (3.89) | 35.19 (3.83) | 7.52 (3.82) | 5.03 (3.85) |
| FR × RR | 7.33 (3.88) | 14.34 (3.83) | 10.00 (3.86) | 1.94 (3.80) | 1.46 (3.88) | 9.87 (3.81) | 12.67 (3.89) | 10.46 (3.87) |
| RF × RR | 7.06 (3.89) | 9.48 (3.82) | 11.25 (3.80) | 3.68 (3.81) | 0.17 (3.83) | 4.86 (3.86) | 6.44 (3.86) | 4.65 (3.81) |
| FF × FR × RF | 9.73 (3.81) | 0.26 (3.85) | 1.01 (3.84) | 17.42 (3.82) | 7.37 (3.79) | 1.84 (3.83) | 6.45 (3.91) | <0.01 (3.86) |
| FF × FR × RR | 1.57 (3.78) | 8.38 (3.90) | 6.48 (3.84) | 8.98 (3.76) | 0.51 (3.82) | 2.36 (3.82) | 4.50 (3.84) | 1.42 (3.85) |
| FF × RF × RR | 6.43 (3.84) | 25.35 (3.82) | 15.46 (3.90) | 11.79 (3.85) | 4.38 (3.81) | 12.05 (3.89) | 0.30 (3.84) | 3.09 (3.89) |
| FR × RF × RR | 4.96 (3.80) | 9.24 (3.79) | 7.18 (3.83) | 1.73 (3.82) | 0.52 (3.81) | 5.92 (3.86) | 7.65 (3.83) | <0.01 (3.87) |
| FF × FR × RF × RR | 6.03 (3.82) | 5.21 (3.81) | 2.51 (3.78) | 2.39 (3.82) | 1.69 (3.87) | 8.77 (3.83) | 26.43 (3.87) | 3.68 (3.81) |

**Supporting Table S1**
