# Supplementary material for: The Effects of Dominance on Leadership and Energetic Gain: A Dynamic Game between Pairs of Social Foragers
Source: PLoS Comput Biol. 2011 Oct 20;7(10):e1002252. doi: 10.1371/journal.pcbi.1002252 (PMC3197661; doi:10.1371/journal.pcbi.1002252)
Supplement: Table S2 — F values and significance terms for results presented in Table 3 . Data are presented in an identical format to Supporting Table S1. (DOCX) [file pcbi.1002252.s002.docx]

| **Subordinate pays extra cost when** | **likelihood dominant repeats behaviour** | **likelihood subordinate repeats behaviour** | **length of time a paired behaviour is repeated** | **energetic reserves of dominant** | **energetic reserves of subordinate** | **length of time dominant heaviest** | **length of time subordinate heaviest** |
| --- | --- | --- | --- | --- | --- | --- | --- |
| both players forage (FF) | 187.07 (3.79) | 1260.20 (3.85) | 0.28 (3.80) | 141.70 (3.80) | 453.35 (3.83) | 0.46 (3.88) | 270.03 (3.77) |
| the dominant forages, and the subordinate rests (FR) | 0.37 (3.84) | 166.37 (3.86) | 13.27 (3.87) | 113.23 (3.89) | 128.31 (3.82) | 18.19 (3.89) | 10.84 (3.84) |
| the dominant rests, and the subordinate forages (RF) | 2.70 (3.86) | 41.70 (3.89) | 5.35 (3.82) | 15.78 (3.89) | 420.34 (3.84) | 136.23 (3.87) | 16.01 (3.82) |
| both players rest (RR) | 292.42 (3.87) | 337.69 (3.86) | 26.66 (3.87) | 41.41 (3.80) | 888.39 (3.80) | 2.09 (3.84) | 290.21 (3.86) |
| **interaction terms** |  |  |  |  |  |  |  |
| FF × FR | 0.35 (3.80) | 2.76 (3.85) | 1.36 (3.83) | 28.18 (3.85) | 77.08 (3.88) | 14.32 (3.82) | 0.04 (3.83) |
| FF × RF | 20.69 (3.87) | 15.22 (3.85) | 7.82 (3.84) | 2.78 (3.83) | 13.34 (3.81) | 8.57 (3.87) | 2.29 (3.87) |
| FR × RF | 0.39 (3.80) | 8.07 (3.86) | 0.02 (3.89) | 6.90 (3.88) | 5.83 (3.83) | 17.25 (3.85) | 1.29 (3.84) |
| FF × RR | 26.56 (3.86) | 9.29 (3.81) | 1.36 (3.89) | 2.75 (3.85) | 2.91 (3.83) | 23.34 (3.78) | 108.70 (3.87) |
| FR × RR | 0.44 (3.90) | 12.58 (3.88) | 1.97 (3.83) | 0.04 (3.78) | 23.20 (3.78) | 6.62 (3.88) | 15.22 (3.81) |
| RF × RR | 0.41 (3.84) | 5.50 (3.92) | 0.17 (3.87) | 2.23 (3.88) | 10.33 (3.81) | 4.81 (3.87) | 0.09 (3.88) |
| FF × FR × RF | 0.18 (3.81) | 0.11 (3.82) | 0.27 (3.83) | 0.16 (3.82) | 2.46 (3.82) | 0.75 (3.78) | 3.58 (3.77) |
| FF × FR × RR | 5.21 (3.87) | 13.15 (3.89) | 3.30 (3.83) | 8.02 (3.80) | 0.87 (3.84) | 0.21 (3.84) | 4.18 (3.76) |
| FF × RF × RR | < 0.01 (3.83) | 4.13 (3.80) | 0.10 (3.81) | 0.67 (3.84) | 1.23 (3.84) | 0.13 (3.84) | 3.11 (3.85) |
| FR × RF × RR | 0.74 (3.83) | 1.15 (3.82) | 0.16 (3.86) | 0.86 (3.84) | 0.03 (3.83) | 0.01 (3.83) | 0.39 (3.86) |
| FF × FR × RF × RR | 0.39 (3.92) | 8.67 (3.87) | 0.13 (3.78) | 0.88 (3.78) | 1.41 (3.84) | 0.03 (3.82) | 0.26 (3.80) |

**Supporting Table S2**
